# Targeting endothelial cell metabolism for cardio-protection from the toxicity of antitumor agents

**DOI:** 10.1186/s40959-016-0010-6

**Published:** 2016-03-15

**Authors:** Lucia Morbidelli, Sandra Donnini, Marina Ziche

**Affiliations:** Department of Life Sciences, University of Siena, Via A. Moro 2, 53100 Siena, Italy

**Keywords:** Nitric Oxide, Vascular Endothelial Growth Factor, Trastuzumab, Sorafenib, Sunitinib

## Abstract

The vascular endothelium plays a fundamental role in the maintenance of tissue homeostasis, regulating local blood flow and other physiological processes. Chemotherapeutic drugs and target therapies, including antiangiogenic drugs targeting vascular endothelial growth factor (VEGF) or its receptors, not only efficiently act against tumor growth, but may also induce endothelial dysfunction and cardiovascular toxicity. Continued research efforts aim to better understand, prevent and mitigate these chemotherapy associated cardiovascular diseases. Conventional chemotherapeutic agents, such as anthracyclines, platinum compounds, and taxanes, and newer targeted agents, such as bevacizumab, trastuzumab, and tyrosine kinase inhibitors, have known risk of cardiovascular toxicity, which can limit their effectiveness by promoting increased morbidity and/or mortality. This review describes a) the activity of anticancer agents in inducing endothelial dysfunction, b) the metabolic pathways and signalling cascades which may be targeted by protective agents able to maintain or restore endothelial cell function, such as endothelial nitric oxide synthase/fibroblast growth factor-2 (eNOS-FGF-2) pathway, and c) the drugs/strategies reported to improve endothelial function and to reduce the risks of cardiovascular diseases such as angiotensin converting enzyme inhibitors (ACEi) and beta blockers, that are fundamental therapies in chronic heart failure (HF), as well as non-standard HF treatments such ad nitric oxide donors and antioxidant strategies. There is increasing interest in whether ACEi, beta-blockers, and/or statins might prevent and/or therapeutically control cardiotoxic effects in cancer patients. Maintaining endothelial function during or following treatments with chemotherapeutic agents, without affecting anti-tumor drug-effectiveness, is essential for preserving or recovering cardiovascular homeostasis. In this respect, the early detection and immediate therapy of cardiovascular toxicity appear crucial for substantial recovery of cardiac function in cancer patients.

## Background

Cardiac endothelial cells (ECs) play a fundamental role in heart function. The myocardium is composed of cardiomyocytes and non-myocytes, fibroblasts and ECs, which work in concert for proper heart functioning [1]. Indeed, while cardiomyocytes generate the contractile force, fibroblasts secrete components of extracellular matrix and paracrine factors, and ECs line the coronary vasculature and allow delivery, via the bloodstream, of the free fatty acids and oxygen required to meet the high metabolic demands of the contractile myocytes [1, 2]. Additionally, cardiac ECs release a glycoprotein, neuregulin-1, that binds to receptor tyrosine-protein kinase ErbB-4 which heterodimerizes with ErbB2. This activates downstream intracellular signalling as the pathways extracellular related kinase1/2 (ERK1/2) and phosphatidylinositol 3-kinase (PI-3 K) involved in contractile function and cardiomyocyte survival and proliferation [3].

**Fig. 1:**
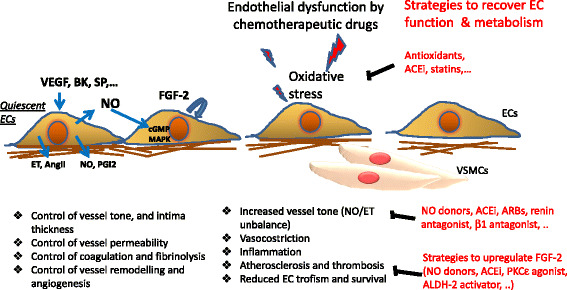
Quiescent endothelial cells (ECs) participate to the physiological maintenance of cardiovascular tissue homeostasis through the control of vessel tone, permeability and intima thickness, coagulation and fibrinolysis, vessel remodelling and angiogenesis. Growth factors as VEGF, or peptides as BK and SP modulate the production/release of vasoactive molecules from ECs, including NO, PGI2, AngII and ET, which, in turn, activate intracellular signalling pathways as MAPK and cGMP pathways, and/or FGF2 production, involved in contractile function and EC survival and proliferation. Endothelial loss-of-function/dysfunction following exposure to conventional chemoterapeutic drugs or target therapies, including VEGF/VEGFR inhibitors. Cancer therapies damage essential signaling cascades that promote undesired cancer cell proliferation, but also protect endothelial cells, especially in response to stress. Endothelial dysfunction is regarded as a decrease of NO released from ECs, an increase of vessel permeability, an increase of platelet adhesion and aggregation, and transmigration of inflammatory cells, which in turn sustain atherosclerosis, vasoconstriction and reduced EC trofism and survival. Endothelial dysfunction is crucial in heart damage. Different categories of drugs have been shown to improve endothelial function and to reduce the risk of cardiovascular diseases associated to treatment with chemotherapeutic agents. Among these, there are ACEi, ARBs, renin and β1 antagonist, NO donor drugs, PKCε agonist and ALDH2 activators. (VEGF: vascular endothelial growth factor; BK: bradykinin; SP: substance P; NO: nitric oxide; ET: endothelin; PGI2: prostacyclin; AngII: angiotensin II; MAPK: Mitogen activated proteine kinase; cGMP: cyclic guanosine monophosphate; FGF2: fibroblast growth factor; VSMCs: vascular smooth muscle cells)

A healthy endothelium is essential for the homeostasis of the whole cardiovascular system. On the other hand, endothelial dysfunction is associated with the pathophysiology of various diseases including atherothrombosis, diabetes, sepsis, pulmonary hypertension, microangiopathies associated with neurodegenerative diseases, liver steatosis and cancer metastasis [4]. Mature ECs, endothelial progenitor cells and circulating ECs participate to the physiological maintenance of cardiovascular tissue homeostasis including vessel tone, permeability and intima thickness, vessel remodelling and angiogenesis, coagulation and fibrinolysis (Fig. 1). Indeed, it has been proposed that endothelial function can be regarded as a “barometer for cardiovascular risk” [5, 6].

## Cardiovascular toxicity by chemotherapeutic drugs

In general, chemotherapy is accompanied by systemic endothelial dysfunction, increasing the cardiovascular disease (CVD) risk and promoting vascular complications [7]. Cancer patients with concomitantly impaired systemic endothelial function may be particularly susceptible to the detrimental effects of anticancer medications. Patients treated with cardiotoxic cancer therapies manifest multiple risk factors such as hypertension, obesity, dyslipidemia and metabolic syndrome, further worsening vascular reserve and resulting in increased risks of cardiotoxicity, which can limit cancer therapies effectiveness by promoting increased morbidity and/or mortality [8].

### Conventional chemotherapy

Cancer therapy-induced endothelial cytotoxicity and cardiotoxicity are due to a combination of “on-target” and “off-target” effects. In particular, cancer therapies may target signaling cascades that promote cancer cell proliferation, but also protect ECs and cardiomyocytes, especially in response to stress (Table 1). The development of molecules/strategies capable of inducing robust anti-tumor responses concurrent with minimal systemic side effects is crucial for the improvement of chemotherapy in oncology.

**Table 1:** Effect of chemotherapeutic agents on various metabolic pathways and functions of human ECs

Drug	Research type	Vascular action
Doxorubicin, daunorubicin	In vitro	Impairment of NO-dependent function DNA damage, ROS generation, caspases 3 & 7 activation, apoptosis
Cisplatin	In vitro	Increased expression of ICAM-1, tPA, PAI-1, CRP, ROS
Paclitaxel, docetaxel	In vitro	Cytoskeleton disruption, impairment of proliferation, migration, prothrombotic effect
5-fluorouracil	In vitro	Blockade of DNA synthesis; disruption of endothelial layer
Trastuzumab	Clinical	Impairment of endothelial NO production, ROS generation
VEGF inhibitors	Clinical	Blockade of VEGF – dependent pro-survival mechanisms in endothelium
		Impairment of endothelial NO production, vasoconstriction, haemorrhages

Mechanisms dependent on reactive oxygen-species (ROS) were among the first to be linked to endothelial toxicity of chemotherapeutics [9] (Fig. 1). Indeed, cardiac and endothelial toxicity of anthracyclines was attributed to redox activation of anthracyclines to semiquinone intermediates, which generate superoxide radicals upon reduction [10]. Both the superoxide anion and its dismutation product - hydrogen peroxide - possess inherent toxicity [11].

Anthracyclines are anticancer compounds that were originally derived from *Streptomyces*. Anthracyclines are red, aromatic polyketides and occur in variety of forms due to the structural differences in the aglycone and the different sugar residues attached [12]. Multiple pathways are thought to be involved in the cytotoxicity of this class of anti-cancer drugs, including accumulation in the nucleus of neoplastic and proliferating cells, DNA intercalation, interaction with/inhibition of DNA binding proteins (such as topoisomerase II-TopII, RNA polymerase, histones), free radical generation, and antiangiogenic mechanism [13].

Anthracycline-induced endothelial toxicity seems to be a complex response, influenced by various mechanisms, including drug-accumulation in nuclei [14] and mitochondria [15], and DNA repair [16], stress-induced signaling mechanisms [17], sarcoplasmic reticulum stress [18], nitrosative stress [19], the activity on drug transporters (including MDR1 and MRP1) [20], drug metabolism [21], and TopI and II inhibition [15, 22].

TopII is a cellular target for anthracyclines [22]. In mammals, there are two isoenzymes of TopII: TopIIa and TopIIb. TopIIa, expressed only in proliferating cells as tumor cells [23], is thought to be the molecular basis for the anthracyclines’ anticancer activity. The ubiquitous TopIIb has elevated expression in terminally differentiated cells, including adult endothelial cells [24]. Thus, TopIIb may directly contribute to the development of anthracycline-induced endothelial toxicity and cardiomyopathy [25]. The observation that pixantrone, a novel anthracycline used in refractory-relapsed non-Hodgkin lymphoma, ineffective on TopIIb, lacked endothelial toxicity and cardiotoxicity, supports the hypothesis that inhibition of TopIIb plays a key role on anthracycline toxicity. However, pixantrone differs from anthracycline for other functional properties potentially associated with its specific toxicity [26, 27].

A better understanding of these mechanisms will help design a rational strategy against the endothelial toxicity of anthracyclines. A liposomal-derived doxorubicin formulation may be used as an alternative, as it is associated with reduced cardiovascular toxicity [28]. Liposomal doxorubicin seems also to be endowed with lower endothelial toxicity, reduced caspase-3 activation and preservation of anti-apoptotic protein Mcl-1 expression in cultured ECs, as compared with doxorubicin [29].

Almost every chemotherapeutics display significant detrimental effects on endothelial function [30]. Cisplatin and most other platinum compounds are simple inorganic molecules containing a platinum ion. Platinum compounds induce tumor apoptosis by activation of signal transduction leading to the death receptor mechanisms as well as mitochondrial pathways. Apoptosis is responsible for the characteristic nephrotoxicity, ototoxicity and most other cytotoxicity of these drugs. Cisplatin-induced cytotoxicity in endothelial cells has been associated with increased formation of pro-coagulant endothelial micro-particles [31] and free radicals [32]. For example, testicular cancer patients treated with cisplatin showed increased plasma levels of the endothelial pro-thrombotic markers PAI-1 and vWF, compared to patients treated only with orchiectomy [33]. Further, Vaughn et al. [34] found that NO-dependent vasodilation (flow-mediated vasodilation) in the brachial artery was impaired in long-term cancer survivors that had received cisplatin-based chemotherapy as compared to chemotherapy-naive patients. These observations suggest that, to protect the EC function, and consequently preserve cardiovascular health, patients treated with alkylating agents such as cisplatin would benefit from association of anti-coagulant or anti-thrombotic drugs [33, 34]. Indeed, recent evidence suggested enhanced platelet activation in tumors (eg, colon cancer), and a reduction in the incidence and mortality for colon cancer in individuals under chronic treatment with low-doses of aspirin, as that recommended for the prevention of atherothrombosis [35]. Ongoing primary prevention and adjuvant trials (eg, ADD-Aspirin Trial) of low-dose aspirin will be of help to investigate the contribution of this strategy on chemotherapy-associated cardiovascular toxicity.

5-fluorouracil (5-FU), a widely used antimetabolite, is a pyrimidine analogue which has been reported to control tumor growth by various mechanisms, including inhibition of thymidylate synthase by 5-fluoro-2′-deoxyuridine-5′-monophosphate, incorporation of 5-fluorouridine-5′-triphosphate into RNA and incorporation of 5-fluoro-2′-deoxyuridine-5′-triphosphate into DNA [36]. In ECs, 5-FU was found to suppress the angiogenic process by blocking the stimulatory effect of vascular endothelial growth factor (VEGF) on DNA synthesis during EC mitosis [37] and to induce ROS-induced endothelial damage [38]. Although inhibition of EC proliferation during tumor angiogenesis is a relevant strategy to starve tumors and decrease their progression, systemic VEGF inhibition also disturbs endothelial cell homeostasis and accelerates atherogenesis and arterial thrombembolic events often resulting in myocardial infarction, cerebrovascular insults, and peripheral or mesenteric ischemia [39–41] (Fig. 1). It may be suggested to accompany treatment with 5-FU or analogous by drugs protecting endothelial cell function, but clinical evidences on the activity of the latter regarding the anti-tumor efficacy of 5-FU are actually not available.

Microtubule-binding drugs, e.g. taxanes, are diterpenes produced by the plants of the genus *Taxus*. Their main mechanism of action involves the inhibition of cell division, chromatid separation and growth, ultimately leading to cell death. These drugs are commonly known as mitotic inhibitors or microtubule inhibitors as they cause a “frozen” mitosis. As with various cancer cells, taxanes impair basic functions also of ECs, such as proliferation and invasion [42]. Paclitaxel also increases endothelial tissue factor (TF) expression via its stabilizing effect on microtubules and activation of c-jun kinase (JNK), thus leading to downregulation of thrombomodulin and increased protein nitration [43]. Another tubulin blocker, vincristine, has been shown to adversely affect rat cardiac microvascular ECs [44]. Endothelial damage has also been reported for other classical chemotherapeutics, including cyclophosphamide (a nitrogen mustard inducing DNA alkylation) [45], bleomycin (anti-tumor antibiotic inducing DNA degradation) and vinca alkaloids (depolarizing agents causing spiral-like distortions of the cellular microtubules) [46]. Taken together, these data suggest that various chemotherapeutics display clinically relevant endothelial damage (Table 1). Accordingly, evaluation of endothelial toxicity of chemotherapeutics could potentially lead to the design/identification of preventive strategies to preserve endothelial function, and, in turn, to reduce the risk for CVDs in long-term cancer survivors without affecting drug-associated anti-tumor efficacy.

### Target therapies

Endothelial damage is also a common feature of various novel biological chemotherapeutics, as anti-VEGF agents (bevacizumab, sunitinib, sorafenib, lapatininb) and anti-Her2 (trastuzumab) [47, 48]. In clinical trials, 25 % of patients receiving trastuzumab developed systolic dysfunction especially when administered with or shortly after doxorubicin [48–51]. Her2 inhibition was also shown to cause impairment of vascular function through a reduction in NO bioavailability and an increase in ROS production [52, 53].

Furthermore, highly effective VEGF inhibitors have also been associated with endothelial toxicity. Anti-VEGF treatment consistently predisposes patients to either thrombosis or bleeding [39], as well as to systemic hypertension, which represents the most common side effect of anti-VEGF treatment [40, 41]. Beside capillary rarefaction, mechanisms leading to endothelial dysfunction and hypertension by VEGF inhibitors are related to reduced NO production, increased ET-1 release and altered renin-angiotensin system [54, 55].

Another mechanism of hypertension may be related to the VEGF-mediated suppression of nephrin, which is important for the maintenance of the glomerular slit diaphragm and may contribute to the proteinuria seen with this class of tyrosine kinase inhibitors [56].

The antiangiogenic multiple kinase inhibitors sunitinib and sorafenib target a range of different receptor tyrosine kinases and other intracellular kinases. Sunitinib and sorafenib, for example, are known to also inhibit platelet derived growth factor receptor (PDGFR), which plays a critical role in angiogenesis and cardioprotection in the setting of pathologic stress. Recent data have suggested a novel mechanism for sunitinib-induced cardiotoxicity, as the drug appears to cause coronary microvascular dysfunction, postulated to be due to loss of pericytes [57]. PDGFR inhibition impairs the growth and survival of pericytes, a cell type closely associated with the microvasculature and supporting the microvascular function of some tissues [58, 59]. Both sunitinib and sorafenib are also known to inhibit the stem cell growth factor receptor known as c-Kit or CD117, which is expressed by precursors of hematopoietic stem cells and endothelial progenitor cells, functioning in the mobilization of these cells to sites of injury [60].

In summary, there is pre-clinical evidence that target therapies for cancer cells frequently affect EC functions, resulting in impaired pro-survival and pro-angiogenic signaling [61]. As reported for conventional chemotherapeutic drugs, the use of target therapy is a beneficial strategy to control tumor progression, but requires the development of new approaches to protect ECs from the direct and/or indirect cytotoxicity of these compounds (Fig. 1).

## Drugs protecting endothelial cell damage associated to chemotherapeutic agents

Different classes of drugs have clearly been shown to improve endothelial function and in some instances to reduce the risk of CVDs associated to treatment with chemotherapeutic agents. Among them, angiotensin converting enzyme inhibitors (ACEi), antioxidants and statins have direct effects on ECs, while angiotensin receptor blockers (ARBs), renin inhibitors, beta blockers, and estrogens indirectly affect EC function (Fig. 1) [29, 47, 62–64]. All these drugs have in common the ability to upregulate the eNOS pathway leading to an increase in plasma NO availability and a general improvement of endothelial function [4].

Among the drugs described below, ACEi and beta blockers are fundamental therapies in chronic heart failure (HF), while NO donors and antioxidant strategies are non-standard HF treatments and represent experimental approaches.

### ACEi

In vivo, the protective effects of ACEi on anthracycline cardiotoxicity are not simply related to hemodynamic effects due to reduced AngII production, like reductions in post-load and persistent mitigation of sympathetic tone [62], but also to direct anti-remodeling, antifibrotic and antioxidant properties which rate this class of drugs as a first line HF therapy [65]. In accordance, ACEi prevent decrements of left ventricular ejection fraction (LVEF) even if they are administered after anthracyclines, cardiomyocyte necrosis and increase of circulating troponin [62]. In long term, inhibition of cardiac remodeling would be important for ACEi to prevent late cardiovascular sequelae. Meta-analysis and retrospective studies on cancer patients treated with anthracyclines, trastuzumab and tyrosine kinase inhibitors who had developed a drop in LVEF, showed that cardioprotective interventions with ACEi, or with beta blockers, statins and ARBs lead to recovery of myocardial function and reduction of cardiac events, thus allowing patients to complete cancer therapy [64, 66]. The temporal indication is that initiation of ACEi and beta blocker treatment should be started as soon as possible after completing chemotherapy [67]. Indeed, no response was observed in patients in whom therapy was initiated >6 months after completion of chemotherapy [68]. The use of ACEi in the prophylaxis of anthracycline-related cardiac dysfunction has been confirmed recently by various authors [69, 70], while their efficacy on trastuzumab treated patients has not firmly been demonstrated, yet [71] and this remains an area of active investigation (NCT00459771, www.clinicaltrials.gov).

The results of prospective studies have been recently published, demonstrating that ACEi pre-treatment or co-treatment of patients suffering of different cancers have a significant preventive effect on cardiotoxicity induced by anthracyclines [72, 73]. Both components of the heart, the microvascular endothelium and cardiomyocytes, are target of the toxic effect of doxorubicin. The number of ECs is 3 fold higher than that of cardiomyocytes, and the vascular component in the heart is crucial for proper functions of cardiomyocytes [1, 2]. Thus, it is important to target the coronary microcirculation to provide benefits to the heart besides the systemic and cardiac effects.

**Fig. 2:**
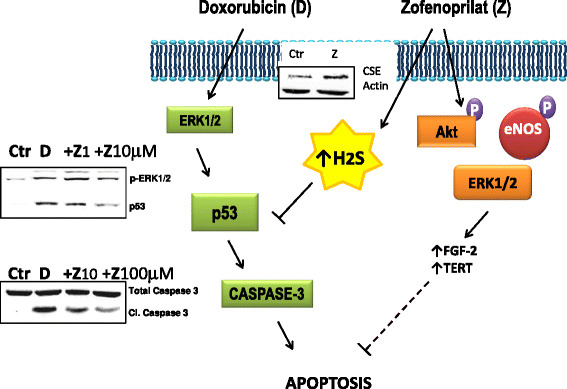
Molecular mechanisms of doxorubicin induced endothelial damage and reversion by the SH-containing ACEi zofenoprilat. Exposure of bovine coronary post-capillary venular endothelial cells to doxorubicin (D) impaired cell survival by promoting their apoptosis (evaluated as cleaved caspase-3: Cl. Caspase 3). ERK1/2 related p53 activation was responsible for doxorubicin induced caspase-3 cleavage. pERK1/2 and p53 were evaluated by western blot in EC treated with 0.5 μM doxorubicin (D) for 1 h, while the cleavage of caspase-3 was monitored by western blotting in EC exposed to doxorubicin for 6 h. P53 mediated-apoptosis and impairment of survival were reverted by treatment with zofenoprilat (Z), added at 1–100 μM concentration together with doxorubicin. The previously described prosurvival signaling pathway (activation of PI-3K dependent eNOS and upregulation of endogenous FGF-2 and telomease reverse transcriptase TERT) [77] was not involved in the protective effect of doxorubicin induced damage, which, instead, could be ascribed to cystathionine gamma lyase (CSE) dependent availability of H_2_S from zofenoprilat. Indeed the levels of CSE protein were upregulated by zofenoprilat treatment (10 μM, 4 h) [78]

The vascular protective properties of ACEi seem related to multiple mechanisms as activation of eNOS (dependent on its turn from bradykinin improved half-life), stimulation of protective intracellular signaling and metabolic pathway, and antioxidant and ROS scavenger properties. Our attention was particularly focused on the sulphydryl group (−SH) containing lipophilic compound, zofenoprilat, the active moiety of the prodrug zofenopril, which, in comparison with other ACEi, is known to accumulate intracellularly [74, 75]. We previously demonstrated a central role of fibroblast growth factor (FGF-2)/FGF receptor-1 system in mediating the acquisition of an angiogenic phenotype in coronary microvascular endothelium [76] by zofenoprilat, reported to exhibit both potent antioxidant and scavenger effects, and anti-inflammatory action [74]. Zofenoprilat, but not other ACEi bearing (i.e. captopril) or not (i.e. enalaprilat) a SH group, was the most potent and effective to promote endothelial cell survival and *ex vivo* angiogenesis [77]. In microvascular endothelium, zofenoprilat up-regulates eNOS, FGF-2 and telomerase (TERT) mRNA, inducing cell survival, rescuing damaged ECs and promoting physiological angiogenesis without synergistic effects with known angiogenic factors produced by tumors as VEGF [76, 77]. Recently, we reported the protective properties of zofenoprilat, known to have an elevated tropism for the heart, against doxorubicin-induced toxicity in coronary ECs [78]. The ACEi zofenoprilat preserves coronary EC survival and function damaged by doxorubicin, and appears to exert its protective effects owing to its SH group, besides the ACE inhibitory function [79]. The presence of a SH group in the zofenoprilat structure suggested us to investigate the involvement of hydrogen sulfide (H_2_S) in the protective effect mediated by zofenoprilat. H_2_S donors induce vasodilatation and vessel remodeling [80], protect ECs from hypoxia/reperfusion damage [81] and exert anti-inflammatory effects in animal models [82]. Animal studies documented the protective effect of the ACEi zofenopril in the reduction of cardiac injury elicited by doxorubicin [73, 83–85], without affecting its antitumor efficiency [84]. Moreover, a protective effect by H_2_S on doxorubicin induced cardiotoxicity has been recently reported [86]. We have demonstrated that H_2_S mediates zofenoprilat activity, since cystathionine γ-lyase (CSE) is upregulated by the drug and CSE pharmacological inhibition prevents zofenoprilat protective effects on doxorubicin induced damage both at a molecular (p53, caspase-3) and a functional level (cell survival) [78] (Fig. 2). The evidence that ACEi protects EC functions potentially suggests that zofenoprilat might also contribute to normalize vessel phenotype at tumor level, thus contributing to maximize cytotoxic drug delivery to tumor cells [87, 88].

Altogether, our data reinforce the indication of the ACEi zofenopril being endothelium protective in patients exposed to the cytotoxic drug doxorubicin.

### Nitric oxide donors

ECs finely control vasomotor responses through the production and metabolism of vasoactive molecules acting on smooth muscle cells, as endothelin-1 (ET-1), nitric oxide (NO), prostacyclin I2 (PGI2), and angiotensin II (AngII) (Fig. 1). Moreover, endothelial cells control vascular permeability, especially in microvascular districts, release molecules that impact on the coagulation and fibrinolytic systems, and synthesize several growth factors, by which ECs interact with circulating cells and support their survival. The most important growth factor produced by ECs is FGF-2, which remains cell-associated or deposited in the extracellular matrix and acts in an autocrine/paracrine manner [89].

Concerning the management of VEGF inhibitors-induced hypertension, there are few case-reports of the efficacy of long-acting NO donor drugs in two patients who were treated with antiangiogenic cancer therapies and were resistant to conventional antihypertensive drugs [90]. Given the hypothesized role of decreased NO signaling in the pathogenesis of hypertension with these agents, NO donor drugs with peculiar and controlled NO release kinetics and able to preserve/restore endothelial survival and function may be a mechanistically relevant class of drugs to use (Fig. 1). However the efficacy of NO donors or drugs able to upregulate eNOS in contributing to vessel normalization without counteracting the antineoplastic/antiangiogenic effect of drugs impairing the aberrant VEGF signaling at tumor level remains to be validated both at the experimental and the clinical level.

**Fig. 3:**
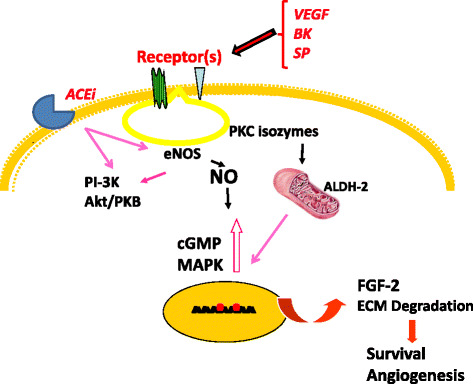
Metabolic pathways preserving EC function. ECs maintain a prosurvival phenotype through the upregulation of eNOS, the activation of cGMP, Akt and MAPK pathways, and the transcription of FGF-2. ACEi, PKCε activators, novel NO donor drugs, VEGF mimetic peptides and ALDH2 activator have been reported to modulate eNOS activity and/or FGF-2 expression, suggesting a potential application of these drugs in association with chemotherapeutic drugs to preserve EC function and to reduce the risk for CVDs in long-term cancer survivors

In this context, we have contributed to demonstrate that microvascular endothelium, in particular from the coronary post-capillary microcirculation, is able to maintain a prosurvival phenotype through the upregulation of eNOS which fine-tunes the release of nanomolar amounts of NO, resulting in activation of cGMP and MAPK pathways, finally controlling the transcription of FGF-2 mRNA at the nuclear level [89, 91–95] (Fig. 3). Indeed, stress insults as serum deprivation and ROS-induced cytotoxic mediators impair the eNOS/FGF-2 pathway [96–98]. Therapeutic strategies able to restore eNOS functioning and FGF-2 production and release have been developed and investigated, as activators of PKCepsilon isoform [99], novel NO donor drugs based on metallic centers [100], a VEGF mimetic peptide named QK [101] and the mitochondrial aldehyde dehydrogenase (ALDH2) activator, called Alda-1 [102] (Fig. 3). Recently, activation of mitochondrial ALDH2 has been also reported to alleviate doxorubicin toxicity [103].

**Fig. 4:**
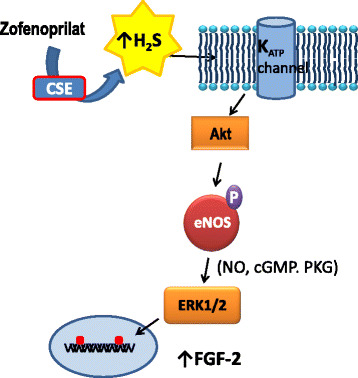
Molecular mechanisms of zofenoprilat-induced protective effects on vascular ECs. Zofenoprilat induces a constant H_2_S production through CSE upregulation in vascular endothelium. On its turn H_2_S maintains ECs survival and stimulates the angiogenic process through a sequential pathway involving K_ATP_ channel/Akt/eNOS/ERK1/2 pathway (for details see [104])

As reported above, the ACEi zofenoprilat exerts endothelium protective properties through off-target mechanisms in endothelial cells, as CSE activation and, in turn, increase H_2_S availability. We have recently reported that ACEi dependent H_2_S exerts protective activity on ECs through activation of K_ATP_ channel, PI-3 K and eNOS, as well as up-regulation of FGF-2 and telomerase, thus maintaining endothelial longevity and function [76–78, 104] (Fig. 4).

In conclusion, these preventive strategies, reported to preserve endothelial cell function, may be useful in association with chemotherapeutic agents to reduce the risk for CVDs in long-term cancer survivors.

### Antioxidants

Clinical use of antioxidants to protect the heart during anthracycline chemotherapy has been controversial due to the potentially reduced cytotoxic efficacy toward cancer cells. Results from randomized clinical trials addressing the issue whether antioxidants either reduce the incidence of clinical heart failure among patients undergoing anthracycline-based chemotherapy or reduce the response rates to anthracycline-based chemotherapy, have been unclear and need to be revised [105]. Studies *in vitro* and in animal models have explored the role of incorporating antioxidants with anthracyclines in the hope of reducing oxidative damage to cardiac cells. Early studies evaluated the effectiveness of common antioxidants found in the diet, which are known to prevent cellular oxidative damage. Many animal models showed a reduction in anthracycline cardiotoxicity by co-administering compounds such as vitamin E, vitamin C, vitamin A, coenzyme Q, and flavonoids. Other pharmaceutical compounds with known antioxidant properties, such as probucol, carvedilol, N-acetylcysteine and dantrolene, have also been found to protect cardiac damage in animal models (see [105] for details).

Consistent with the “iron and free radical hypothesis”, the mechanism of action of the only clinically approved drug to prevent anthracycline-mediated cardiotoxicity, dexrazoxane (ICRF-187), was based on its iron-chelating effects [106]. However, accumulated evidence suggests that ROS-dependent mechanisms cannot fully account for anthracycline toxicity. For example, doxorubicin-induced DNA strand breakage was found to be independent of the induction of oxidative DNA damage, while lovastatin reduced doxorubicin-triggered strand breaks without affecting ROS formation [107]. Furthermore, dexrazoxane appeared to be a catalytic inhibitor of TopII, suggesting that protection against anthracycline-induced toxicity could be exerted also via the TopII inhibition, not only via the iron chelation activity of the drug [108].

Other medications that have antioxidant properties may also be effective therapies for chemotherapy-induced cardiovascular damage. HMG-CoA reductase inhibitors, or statins, have been shown to have pleiotropic effects including antioxidant and anti-inflammatory properties, but clinical data are necessary to confirm their efficacy in preventing cancer therapy-related endothelial and cardio-toxicity [64, 107, 109, 110]. Indeed, antioxidant strategies are not established therapies in this setting [70].

In conclusion, if antioxidants are confirmed to reduce anthracycline-associated toxicity while maintaining the drug-associated-antitumor effect, they would provide a useful tool to prevent cardiovascular damage.

### Drugs affecting endothelial cell metabolism

Healthy ECs generate energy through glycolysis [102, 111, 112], and mitochondria represent essentially a bioenergetic reserve on which they count under stress conditions [113]. Recently, we demonstrated the protective role of mitochondrial aldehyde dehydrogenase-2 (ALDH2) in ECs exposed to stress insult [102]. ALDHs are a family of NADP-dependent enzymes with common structural and functional features that catalyze the oxidation of a broad spectrum of aldehydes. Three major classes of mammalian ALDHs have been identified, named 1, 2 and 3. Classes 1 and 3 contain both constitutive and inducible cytosolic isozymes [114]. Class 2 consists of constitutive mitochondrial isozymes [114].

The ALDH dysfunction has been associated with myocardial infarction and hypertension [115], as well as with doxorubicin-mediated cardiotoxicity [103]. For example, ALDH2 has been reported to play a central role in the vasodilator actions of nitroglycerin, and in protecting ischemic myocardium. By preventing ALDH2 inactivation by stress insult, the ALDH2 selective agonists reduce the extent of infarction-induced injury [103, 115].

Further, experimental data have demonstrated that endothelial progenitor cells expressing high levels of ALDH, isolated from bone marrow or peripheral blood, are critical for vascular recovery of the ischemic regions [111]. Within the pancreas, ALDH expressing cells promote islet revascularization after transplantation into streptozotocin-treated mice [112], suggesting that high expression of ALDH in proangiogenic cells plays a key role in tissue vascularization.

In this context, we showed that in ECs exposed to a cytotoxic insult, by preventing the inactivation of the constitutive mitochondrial ALDH2, we could restore mitochondrial functions and rescue the pro-angiogenic functions [102]. We observed that ALDH2 activation promotes growth of cultured endothelium and its ability to form cord-like structures in vitro, similar to those observed in vivo during tissue revascularization. Specifically, our findings demonstrate that activation of ALDH2 prevents the injurious effects of aldehydes on vascular endothelium, preserving EC-associated responsiveness. These results suggest that the above described molecules may be potentially useful to preserve ECs from damage-associated with the treatment with chemotherapeutic drugs.

However, since high ALDH metabolic activities have been observed not only in ECs but also in tumor cells, and ALDH expression in tumor cells has been reported to confer resistance to chemotherapeutic agents as cyclophosphamide [116], inhibitors of ALDH have been proposed in cancer treatment. As observed for anthracyclines, ALDH inhibition in ECs may damage crucial metabolic signaling cascades that protect ECs and cardiomyocytes, especially in response to stress [103]. In this context, it is essential to understand whether ALDH isozymes in ECs might govern their metabolic activity during angiogenesis, and whether they might be a new anti-metabolic/anti-angiogenic druggable target.

## Concluding remarks

It has become clear that cancer patients should be managed by a team of oncologists and cardiologists, as systemic effects of chemotherapeutic drugs, among which cardiovascular are the most important, may occur more frequently [117].

Drugs shown to directly improve endothelial function and to reduce the risk of CVDs associated to treatment with chemotherapeutic agents are ACEi, antioxidants and statins, while angiotensin receptor blockers, renin inhibitor, beta blockers, and estrogens generally improve cardiovascular function. Recently, beta blockers have been demonstrated to be important as ACEi in HF from antineoplastic drugs [68]. The use of drugs that control platelet aggregation and coagulation is also recommended.

There is the need for evaluating cardiotoxicity of potential novel chemotherapeutic agents through specific screening modalities [118] and endothelial cytotoxicity in vitro and in animal models [119], before any drug is approved in clinical trials. The above data show that drug-induced cardiac toxicity can have a multicellular component, in particular most of chemotherapeutic drugs target ECs. This has profound implications for the development of in vitro preclinical cardiovascular toxicity screens within the pharmaceutical industry, based on detecting adverse effects in cardiomyocytes. There is a need for multicellular models (e.g. co-cultures) that reconstitute cardiac physiology to allow researchers to simultaneously investigate structural and functional changes in different cardiac cell types. As an example, the use of Zebrafish comes in help to study the formation of the vertebrate vascular network. The small size and optical translucency of the zebrafish embryo, in combination with huge amounts of fluorescent transgenic lines which allow direct visualization of in vivo vessel formation during embryogenesis, have greatly advanced our understanding of vascular biology and should help us to investigate the cardiovascular toxicity of chemotherapeutic drugs [120].

There is the medical need to identify patients at risk of developing cardiotoxicity as early as possible before and during chemotherapy by the definition of validated biomarkers of vascular and cardiac toxicity [70, 117, 121, 122]. Novel potential robust approaches to address this issue include measurement of circulating miRNAs [123] and the use of metabolomics to identify metabolic signatures in the circulation which are indicative of endothelium injury. These may help to detect changes of cardiotoxicity [124].

Endothelial metabolism and signalling pathways such as eNOS, FGF-2 or ALDH activity may be novel targets for drugs with a protective effect against the cardiovascular toxic effects of chemotherapeutic agents. Further, identification of metabolic determinants governing the metabolic shift of endothelial cells during tumor angiogenesis, might also allow to identify new anti-metabolic/anti-angiogenic targets to be exploited in cancer treatment.

## Abbreviation

5-FU: 5-fluorouracil
ACEi: angiotensin converting enzyme inhibitors
ALDH: aldehyde dehydrogenase
AngII: angiotensin II
ARBs: angiotensin receptor blockers
BK: bradykinin
cGMP: cyclic guanosine monophosphate
CRP: C reactive protein
CSE: cystathionine γ-lyase
CVD: cardiovascular diseases
ECs: endothelial cell(s)
eNOS: endothelial nitric oxide synthase
ErbB-2/-4: receptor tyrosine kinase-2/-4
ERK1/2: extracellular related kinase1/2
ET-1: endothelin-1
FGF-2: fibroblast growth factor-2
H2S: hydrogen sulfide
HF: heart failure
HMG-CoA: 3-hydroxy-3-methylglutaryl-coenzyme A
ICAM-1: intercellular adhesion molecule 1
JNK: c-Jun kinase
LVEF: left ventricular ejection fraction
MAPK: mitogen activated protein kinase
Mcl-1: myeloid cell leukemia-1
MDR1/MRP1: multidrug resisance-1/multidrug resistance protein-1
NO: nitric oxide
PAI-1: plasminogen activator inhibitor-1
PDGFR: platelet derived growth factor receptor
PGI2: prostacyclin I2
PI-3 K: phosphatidylinositol 3-kinase
PKC: protein kinase C
PKG: protein kinase G
ROS: reactive oxygen species
SP: substance P
TERT: telomerase reverse transcriptase
TF: tissue factor
TopI/II: topoisomerase I/II
tPA: tissue plasminogen activator
VEGF: vascular endothelial growth factor
VSMCs: vascular smooth muscle cell(s)
vWF: von Willebrand factor
